# Liver Damage and COVID-19: At Least a “Two-Hit” Story in Systematic Review

**DOI:** 10.3390/cimb45040199

**Published:** 2023-04-04

**Authors:** Michele Montori, Gialuca Svegliati Baroni, Pierangelo Santori, Catia Di Giampaolo, Francesca Ponziani, Ludovico Abenavoli, Emidio Scarpellini

**Affiliations:** 1 Transplant and Hepatic Damage Unit, Polytechincs University of Marche, 60121 Ancona, Italy; 2 Hepatology and Internal Medicine Unit, Madonna del Soccorso General Hospital, 00168 San Benedetto del Tronto, Italy; 3 Digestive Disease Center (C.E.M.A.D.), Fondazione Policlinico Universitario Agostino Gemelli IRCCS, 00168 Rome, Italy; 4 Translational Medicine and Surgery Department, Università Cattolica del Sacro Cuore, 00168 Rome, Italy; 5 Department of Health Sciences, University Magna Græcia, 88100 Catanzaro, Italy; 6 Translational Research Center for Gastrointestinal Disorders, Gasthuisberg University Hospital, KULeuven, 3000 Lueven, Belgium

**Keywords:** liver disease, COVID-19, acute liver damage, drug-induced liver injury, antivirals

## Abstract

COVID-19 pandemic waves have hit on our lives with pulmonary and, also, gastrointestinal symptoms. The latter also includes acute liver damage linked to direct SARS-CoV-2 action and/or drug-induced (DILI) in the frame of pre-existing chronic liver disease. We aimed to review literature data regarding liver damage during COVID-19. We conducted a systematic search on the main medical databases for original articles, reviews, meta-analyses, randomized clinical trials and case series using the following keywords and acronyms and their associations: liver disease, COVID-19, acute liver damage, drug-induced liver injury, antivirals. Acute liver damage due to SARS-CoV-2 infection is common among COVID-19 patients and is generally self-limiting. However, chronic hepatic diseases, such as metabolic-associated fatty liver disease (MAFLD), are associated with a less favorable prognosis, especially when alkaline phosphatases show a significant rise. Pathophysiology of COVID-19 liver damage is multifaceted and helps understand differences in liver derangement among patients. Thus, early recognition, monitoring and treatment of liver damage are crucial in these patients. In the frame of a not-ending pandemic sustained by SARS-CoV-2, it is crucial to recognize acute hepatic decompensation due to the virus and/or drugs used for COVID-19 treatment.

## 1. Introduction

Although humanity has met with numerous epidemics and also pandemics that have affected millions of lives, our resilience capability has never been trained and challenged. In fact, the SARS-CoV-2 pandemic has terribly conditioned our life in the past three years. This new pathogen has posed a threat not only to our lives but also to global economic security and, last but not least, our healthcare system.

Severe acute respiratory syndrome coronavirus-2 (SARS-CoV-2) is a novel coronavirus that was first identified in Wuhan, Hubei, China, and is responsible for the multi-systemic hyper-inflammatory syndrome COVID-19. In detail, SARS-CoV-2 is the seventh coronavirus known to infect humans [1,2]. Its emergence has been made possible by more and more frequent cross-species infections and occasional spillover events due to globalization [2]. Two of these coronaviruses were responsible for major epidemics in the past two decades: severe acute respiratory syndrome coronavirus (SARS-CoV) (China in 2002–2003) and Middle East respiratory syndrome coronavirus (MERS-CoV) (started and contained in the Middle East in 2012 [3]. The SARS-CoV epidemic was fought and contained through the efforts and sacrifice of Italian researcher Carlo Urbani [4]. All three of these coronaviruses are considered to have a zoonotic origin and have the ability to cause severe and fatal illnesses in humans [5,6]. The issue in their management is due to difficulties in building up an efficient vaccine. In fact, their large genetic diversity, frequent genome recombination and increased human–animal interface activities due to modern agricultural practices sustain novel coronavirus evolution and perpetuate periodic seasonal spreads [4,5].

COVID-19 symptoms commonly occur within 4–5 days from SARS-CoV-2 exposure. However, the window period can be shorter, especially with the last virus variants [7,8]. The most frequent symptoms reported in the literature are fever, cough, fatigue and shortness of breath, which are very similar to other seasonal cases of flu [6,7]. Indeed, other symptoms have been variously represented during the different pandemic waves. Among these, we must mention gastrointestinal symptoms characterized by nausea, vomiting and diarrhea [9]. Indeed, liver damage is also present and is represented by liver enzyme elevation. Usually, this rise is mild and self-limiting. In a few cases, acute liver failure starts and can overlap with drug-induced liver damage and pre-existing chronic liver diseases [10,11].

Thus, we aimed to review literature data on liver manifestations of COVID-19, its pathogenesis and pathophysiology and its interaction with drug-induced liver damage (DILI) and chronic liver disease.

## 2. Materials and Methods

We conducted a systematic PubMed and Medline search for original articles, reviews, meta-analyses and case studies using the following keywords, their acronyms and their associations: liver disease, COVID-19, acute liver damage, drug-induced liver injury, antivirals. When appropriate, preliminary evidence from abstracts belonging to the main national and international gastroenterological meetings (e.g., United European Gastroenterology Week, Digestive Disease Week) was also included. The papers found from the above-mentioned sources were reviewed by two of the authors (E.S. and M.M.) according to PRISMA guidelines [12]. The last Medline search was dated 31 December 2022. The systematic review was registered in PROSPERO (number 405056).

**Figure 1:**
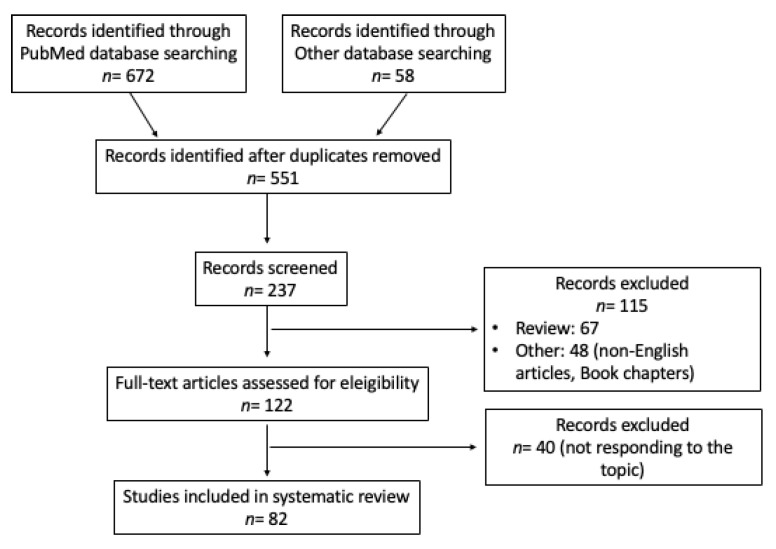
Scheme of record selection according to PRISMA guidelines.

In detail, we found 730 manuscripts matching our search according to keyword combinations from PubMed and other databases. Subsequently, we removed 179 articles because of duplication and after reference checking. After the screening, 237 papers were accepted. We removed 115 papers from the screened group because they were not in English, were a review or book chapter or had non-matching keyword combinations. The remaining 122 papers were checked for eligibility, and we accepted 82 manuscripts. We excluded 40 papers as they did not fully match the topic (Figure 1).

Finally, in our review process, we included data from 30 original articles (including animal studies), 20 RCTs, 31 reviews of the scientific literature, 8 systematic reviews and from 4 metanalyses (Figure 1).

## 3. Results

### 3.1. COVID-19 and Liver Involvement: The Issue

Hepatic damage in COVID-19 patients, regardless of pre-existing liver disease, is defined as “COVID-19-related liver injury” [13]. In fatal cases of COVID-19, liver injury estimated incidence ranges between 58% and 78% [14]. Reversible liver damage (characterized by a short-term mild elevation of liver enzymes) incidence has an almost ubiquitous trend in mild COVID-19 [15]. Men have a higher risk of liver injury development; children have a lower risk than adults [16].

Characteristically, COVID-19-related liver damage is essentially hepatocellular vs. cholangiocellular [6]. This point is interesting and apparently controversial: the site of SARS-CoV-2 entrance into the cell, angiotensin-converting enzyme 2 (ACE2), is significantly more expressed in the cholangiocytes vs. hepatocytes [17]. Clinically, jaundice presentation and elevated total bilirubin levels are also very rare findings in COVID-19 patients [18]. Besides elevated liver enzymes, scientific literature, newspaper and press release have shown cases of darkened face and pigmentation related to liver injury in patients recovering from severe COVID-19 [19]. These features of hepatic damage have been mainly attributed to increased circulating levels of melanin and the elevated iron levels of these patients [8].

Intriguingly, postmortem examination of liver tissue biopsy showed microvascular steatosis and inflammatory liver lobular and portal activities [20]. Moreover, they have been observed: hepatomegaly, pigmented (red) hepatocytes degeneration, neutrophil infiltration and tubular degeneration [9]. Therefore, the entrance of monocytes and lymphocytes in the portal area, together with clogging of hepatic sinus micro-thrombosis, have also been observed with no evidence of bile duct involvement [9].

From a laboratory point of view, COVID-19 liver damage is characterized by higher values of alanine aminotransferase (ALT) and aspartate aminotransferase (AST) and, in the cases of bile duct involvement, by cholestasis features (namely, increased alkaline phosphate (ALP) and gamma-glutamyl transferase (GGT)), as recorded both in Wuhan and during the later pandemic waves in China [21,22].

Very commonly, higher levels of AST, ALP and ALT appear to be the most prevalent finding for physicians at admission [23]. Less frequently, we can find higher values of total and direct bilirubin and reduced values of synthetic liver capability (e.g., reduced total albumin values and higher prothrombin time). These findings do not differ between men and women with COVID-19 [11].

However, increased AST and ALP levels are significantly and specifically associated with COVID-19 liver injury vs. derangement of ALT and total bilirubin values [9,10,11].

Lactate dehydrogenase occurs significantly higher in severely ill patients, although COVID-19 liver injury mostly presents as mild disturbance of liver function tests [24].

In pediatrics, COVID-19 liver injury was associated with mild haemolytic aneamia, elevated lactate dehydrogenase, elevated anti-liver-kidney-microsomal antibody (anti-LKM) and undetectable haptoglobin [25].

In liver cirrhosis patients, other observed altered laboratory tests are increased levels of conjugated bilirubin, hyperammonemia, hypoalbuminemia and worsening coagulopathy [13].

Interestingly, elevated blood levels of aminotranspherases have been explained as a cross-reaction with those of pro-inflammatory cytokines as inducible protein (IP)-1, granulocyte colony-stimulating factor (GM-CSF), monocyte chemoattractant protein (MCP), IFN-γ and macrophage. This cytokine storm is significantly increased in fatal COVID-19 patients with liver derangement [26].

### 3.2. COVID-19-Related Liver Damage: Pathophysiology

Several mechanisms have been associated with the development of COVID-19-associated liver injury.

First, it can be reassembled as hypoxic hepatitis determined by changes in the hemodynamics and oxygen delivery to liver tissue [10]. Interestingly, this is reflected by a sharp rise in aminotransferases and predisposes to respiratory failure, shock and/or cardiac failure.

Furthermore, COVID-19 liver injury can be caused by hepatic ischemia and venous congestion due to elevated venous pressure [10].

Second, SARS-CoV-2 infection is characterized by increased immune response activity. In fact, immune activity in the liver itself is increased in COVID-19 patients. In detail, the number of liver macrophages significantly increases according to the hyper-production of pro-inflammatory cytokines, such as c-reactive protein (CRP), serum ferritin, lactate dehydrogenase (LDH), D-dimer, interleukin (IL)-6 and IL-2. Further, there is increased activity of cytotoxic T-cells with consequent liver tissue damage [27,28]. From a molecular point of view, in more detail, the cytokine storm is characterized by increased production of IL-2, IL-7, IL-10, granulocyte colony-stimulating factor (G-CSF), monocyte chemoattractant protein (MCP1), macrophage inflammatory protein-1 alpha (MIP1A), tumor necrosis factor (TNF), CXC-chemokine ligand-10 (CXCL-10) and C-reactive protein [27,28]. Within the liver, hepatic stem cells (HSCs) respond to this inflammatory load with transdifferentiation into collagen-producing myofibroblasts (MFB). The latter is characterized by the expression of α-SMA (alpha-smooth muscle actin) and the deposit of large amounts of extracellular matrix (ECM). However, HSCs produce themselves pro-inflammatory cytokines (e.g., interleukin IL-1β and IL-18) [29]. Furthermore, HSCs have a fibrogenic action because of cytokine storms. Hepatic fibrogenesis is due to fibrogenic signaling, chemokines production, adipokine secretion, neuroendocrine axis molecules, angiogenesis initiation and NADPH oxidase/oxidant stress [30]. In fact, HSCs express several chemokine receptors (namely, CXCR3, CCR5, and CCR7) and secrete themselves chemokines (namely, CCL2, CCL3, CCL5, CXCL1, CXCL8, CXCL9, and CXCL10) [31]. These chemokines promote fibrogenic cell migration to sites of injury, producing inflammation amplification, specifically through the CCR5 binding RANTES receptor. The activation of the latter results in the migration and proliferation of HSC [32].

Third, direct SARS-CoV-2 infection of liver cells can determine liver injury [8]. Importantly, the liver is one of the human body organs with a higher SARS-CoV-2 infection rate. Moreover, the presence of the angiotensin-converting-enzyme-2 (ACE2) receptors in the bile duct also create the condition for the risk of bile duct dysfunction. The latter, in turn, affects liver regeneration and immune response [33]. In more detail, the entrance of SARS-CoV-2 into these two subsets of cells happens through the interaction of ACE2 and spike (S) protein receptor for SARS-CoV-2 with cleaving of S protein transmembrane serine protease 2 (TMPRSS2) to ACE2 [34].

Fourth, the gut–liver axis is deranged in liver damage in COVID-19 patients. The gut–liver axis is a physiological and also pathophysiological model linking gut and liver functioning through gut microbiota interplay with these two human body districts [35]. In fact, ACE2 is highly expressed in small bowel enterocytes, and their infection by SARS-CoV-2 can benefit the portal circulation to enter the liver via the reticular system [36]. Indeed, SARS-CoV-2 viral RNA has also been detected in fecal samples [37]. From a therapeutic point of view, the use of probiotics and postbiotics to modulate the inflammatory response of COVID-19 patients and reduce viral particles entrance into human body cells has shown promising results and confirmed gut–liver axis involvement and derangement in these patients [38,39].

**Figure 2:**
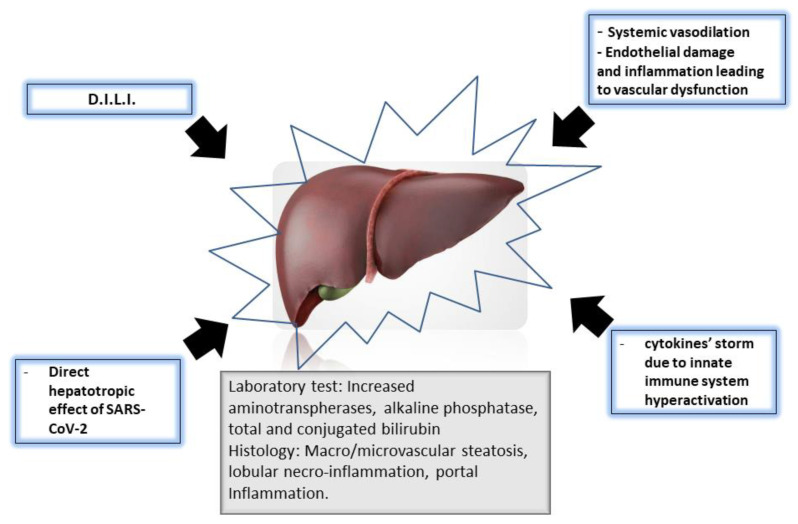
Pathophysiological mechanisms of coronavirus disease 2019 (COVID-19) liver injury. Legend: DILI, drug-induced liver injury; SARS-CoV-2, severe acute respiratory syndrome coronavirus. A special section is due to drug-induced liver damage (DILI) during COVID-19 treatment.

Finally, acute-on-chronic liver failure is the fifth mechanism involved in COVID-19 liver damage [40]. In specific, patients with chronic liver diseases are prone to liver damage development during SARS-CoV-2 virus infection. Moreover, drugs used in COVID-19 patients, such as tocilizumab and baricitinib, are able to lead to hepatitis-B-virus (HBV) reactivation with liver derangement [41]. On the other hand, it is a field of debate on the role and impact of SARS-CoV-2 infection on cholestasis worsening in cholestatic disease (e.g., primary biliary cholangiopathies, sclerosing cholangitis) patients [42] (Figure 2).

### 3.3. Drugs’ Role in Liver Damage in COVID-19 Patients

Drug-induced liver injury is defined as hepatic tissue damage due to direct drugs’ effect or its metabolism within the liver and hypersensitivity to the medication [43]. DILI is classified according to the duration of injury (namely, acute or chronic) and to the histological site of damage (e.g., cholestatic, hepatocellular and/or mixed pattern) [9]. 

In the case of CVOID-19 patients, DILI can overlap with those caused by direct and indirect SARS-CoV-2 infection and can result from the drugs used in patient treatment that varied significantly among pandemic waves. Firstly, it was considered responsible for liver failure; the use of angiotensin II receptor blockers and ACE inhibitors can cause liver failure [44].

Pathophysiologically, the association between COVID-19 and DILI can also be explained by fat deposition (namely, steatosis) induced by medications, such as sodium valproate, amiodarone, tamoxifen and methotrexate [40]. As an example, tamoxifen, an estrogen antagonist, has been found in hepatocellular carriers because of hepatotoxicity mechanisms (e.g., reduced fatty acid beta-oxidation and consequent NASH) in women aged 50–70 years with a history of mastectomy, diabetes, hysterectomy, high cholesterol, high blood pressure and osteoporosis [45].

Mainly, we can list antiviral agents (e.g., remdesivir), nonsteroidal anti-inflammatory drugs (NSAIDs), hydroxychloroquine, antibiotics and last but not least, herbal medications, especially in the Asiatic world [8,46] (Table 1).

Indeed, the main drugs’ metabolism occurs in the liver, with a constant risk for COVID-19 patients, often treated with multi-medications or step-wise drugs.

Thus, strict monitoring of potentially hepatotoxic drugs in COVID-19 patients has to be preferred. A higher probability of DILI occurrence is sustained by older age and sex (namely, men), chronic alcohol consumption, and pre-existing liver disease [54]. Meta-analysis data show a pooled prevalence of DILI of 25.4% in the course of COVID-19 [55]. Part of these patients is represented by severe COVID-19 cases, exposed for a long time to several drugs during hospitalization.

Histologically, mild lobule and/or portal region inflammation and moderate microvesicular steatosis represent COVID-19 DILI features.

In detail, immunosuppressants (e.g., tocilizumab and dexamethasone) have been first linked to DILI cases [9,10,11]. In fact, COVID-19 patients treated with tocilizumab reported serum transaminases increased by 40-fold, eventually resolved within 10 days [56]. Moreover, immunosuppressants can reactivate occult hepatitis B-virus [13] (Table 1).

During the first wave of the pandemic, the use of antiviral drugs (namely, lopinavir and ritonavir) was associated with adverse liver effects, as confirmed by a randomized controlled trial [57]. Further, in 148 patients evaluated in a retrospective manner, lopinavir/ritonavir administration was identified as an independent risk factor for severe liver injury in COVID-19 patients [58].

Among antibiotics, azithromycin was associated with cholestatic hepatitis within 3 weeks upon treatment start [59] (Table 1).

### 3.4. COVID-19, Liver Damage and Chronic Liver Disease: The MAFLD and Other Cases

Chronic liver disease of viral (namely, B and C chronic infections), metabolic, alcoholic, autoimmune origin can be regarded as deterioration of liver function, characterized by a progressive inflammation of the liver tissue and by its destruction and regeneration, occurring in at least a 6-month period. Altogether, this results in liver fibrosis until cirrhosis develops [60].

In detail, COVID-19 patients show a low prevalence of chronic liver disease, ranging from 3 to 8% [61]. Indeed, what counts about having a pre-existing liver disease, is the different impact of liver damage on SARS-CoV-2 infection and vice versa. In fact, several studies have been performed to describe this fine interaction. For example, in a multinational retrospective cohort study including more than 220 patients across 13 Asiatic countries, pre-existing liver disease is shown to be worsened by SARS-COV-2 infection in a bi-directional way [62]. Even more, drug-induced hepatotoxicity of medications for COVID-19 management can confound the field.

Special attention deserves the metabolic associated fatty liver disease (MAFLD) patients. Indeed, the risk of severe COVID-19 is higher in persons with pre-existing MAFLD [63,64]. It is interesting to note that although the MAFLD definition includes conditions that, independently, are risk factors for worse COVID-19 evolution (e.g., obesity, type 2 diabetes), non-alcoholic fatty liver disease (NAFLD) (according to the traditional fatty liver disease definition, characterized by liver steatosis only) itself is an independent risk factor for severe SARS-CoV-2 infection [65]. Further, in 214 Chinese patients, multivariate analysis adjusted for age, sex, smoking, diabetes, hypertension and hyperlipidemia, MAFLD and obesity only were associated with increased severity of COVID-19 infection [66]. In specific, COVID-19 severity is greater in non-alcoholic steatohepatitis (NASH) patients with severe liver fibrosis vs. those with a mild one [55].

From a hepatologic point of view, NAFLD presence is a predictor of COVID-19-associated liver injury [67]. Pathophysiologically, liver damage due to MAFLD pre-existence is not associated with ACE-2 hyper-expression. In fact, MAFLD pathophysiology is associated with increased toll-like receptor (TLRs) expression within the liver and adipocytes and, Kupffer cells have a hyper-secretion of pro-inflammatory cytokines that lead to a chronic micro-inflammatory state exacerbated by insulin resistance and free fatty acids income from inflamed adipose tissue [55,61]. Thus, there are macrophage hyper-activations within the liver with innate immune system dysfunction due to the hyper-representation of immuno-suppressor M2 macrophages [61]. Finally, MAFLD patients have a constant rise in insulin levels, which is significantly correlated with decreased respiratory capacity in COVID-19 [55,61]. In conclusion, it is worth mentioning that NAFLD progresses to NASH in long-term COVID-19 [68].

The case of HBV chronic infected patients under treatment is of interest. The severity of COVID-19 seems not to be affected by chronic HBV infection [69]. On the other side, patients with chronic B hepatitis have a higher risk of developing COVID-19 vs. healthy subjects [70]. However, COVID-19 patients with HBV infection have an increased risk of liver damage and hepatitis B infection reactivation, also explained by immunosuppressants use in the frame of COVID-19 treatment. Interestingly, this special subset of patients presents with a characteristic rise in total bilirubin levels and has a higher mortality rate [60].

Because of liver cirrhosis-related altered immune system functioning, characterized by the micro-inflammatory response within the liver and affecting gut permeability with a tendency towards immune tolerance depression for hepatocytes presenting with oncogenic features, severe acute respiratory syndrome (SARS) from SARS-CoV-2 infection has a higher prevalence in patients with liver cirrhosis [71]. High SARS incidence in cirrhotic patients significantly correlates with the development of consensual liver damage (namely, acute-on-chronic liver failure) [72]. The latter is mainly related to hypoxic stress and/or sepsis development [73]. Further, liver cirrhosis is associated with an increased mortality rate in patients with acute respiratory distress syndrome (ARDS) and also COVID-19. The latter needs further data to be confirmed [74].

Data on the severity of COVID-19 in HCC patients is very limited. Indeed, COVID-19 has a negative impact on cancer patients in general [75]. In detail, the American Association for the study of liver disease (AASLD) recommendations underline the fact that HCC, as a chronic liver disorder, can take up to two months to recover completely from COVID-19 [76]. More in particular, Zhang et al. studied a sample of 28 patients and found those with cancer and, in detail, those with HCC had a significantly lower clinical condition vs. those without cancer [77]. In fact, the immune system surveillance of these patients is depressed, also because of malnutrition and subsequent anemia, hypoproteinemia. Altogether, these factors can favor infection by SARS-CoV-2 [78].

A special mention is deserved to liver transplanted patients. It is well-recognized that liver transplant recipients are more vulnerable to SARS-CoV-2 infection and have a higher potential for virus shedding. In fact, liver-transplanted patients account for almost 4 million individuals because liver transplantation is the world’s second most prevalent solid organ transplant [11]. Although there is the potential for virus passage from donor to recipient, the molecular mechanism of this passage has not yet been discovered. However, there is strong evidence showing that transplanted patients have a lower mortality rate vs. non-transplanted ones. This is explained by a modulated immune system response and lowered “cytokines’ storm” by immunosuppressant drugs [11,77]. Molecularly, immunomodulators used for transplanted long-term maintenance are able to down-regulate the second phase of SARS virus infections. In fact, in this step, there is a significant CD4+ T cell count drop, and CD8+ T cell and macrophages reduced function with cytokine cascade and subsequent COVID-19 exacerbation [78]. Whenever this storm is inhibited by immunosuppressors, there is an elevated viral load with a prolonged disease that can cause increased disease severity in transplant recipients [11,78].

Indeed, the issue of liver transplant recipients is multifaceted. In fact, abuse of medications, in general, and the use of immunosuppressive drugs, in particular, has been associated with liver transplantation. In detail, when the administration of hydroxychloroquine, azithromycin is operated together with calcineurin inhibitors (CNI) and mammalian target of rapamycin (mTOR) inhibitors, levels of these should be controlled [11,73]. In addition, calcineurin and mTOR inhibitors can affect the severity of COVID-19 disease. In liver transplants, tacrolimus levels can be significantly elevated by chloroquine co-administration [11,77,78].

### 3.5. Liver Damage Management during COVID-19

Usually, COVID-19-associated liver injury is transient and self-limiting. Clinicians must pay particular attention to patients with a history of pre-existing liver disease (chronic viral hepatitis and chronic alcoholic liver disease, perhaps at the cirrhosis stage) and transplant recipients. These two subsets of patients must be closely monitored. In detail, ALP peak values are predictive of a worse prognosis [79]. Thus, routine laboratory monitoring of liver function can allow physicians in the early recognition of acute liver injury during COVID-19 in these patients and start a prompt implementation of hepatic function. Interestingly, we must recognize that transaminase elevation of one- to two-fold seems to be a liver protective cut-off vs. normal levels; in particular, this mild rise seems to be associated with liver protection from ischemic or hypoxic damage [55,61,80].

In the frame of drug-induced liver injury during the SARS-CoV-2 infection, identification and withdrawal of any hepatotoxic drug are crucial to patient management. Meanwhile, patients can have a therapeutic switch to more suitable alternatives [81].

Whenever supportive therapy is necessary for the treatment of acute viral and/or DILI or acute-on-chronic liver failure, we can list its steps as substitution of offending drugs, treatment of cirrhosis-related decompensation feature (e.g., hepatic encephalopathy with rifaximin, lactulose and branch chain amino acids and paracentesis for ascites) and supportive therapy initiation with immunosuppressants and anti-inflammatory drugs (e.g., methylprednisolone) [82].

## 4. Conclusions

COVID-19 has conditioned our lives during the last 3 years. The end of this pandemic is still without a precise date. The clinical and laboratory alterations of liver involvement during COVID-19 are subtle and unpredictable, even in the absence of pre-existing liver disease. Chronic liver disease, in particular NAFLD and MAFLD, predisposes patients to worse liver damage progression. However, the natural course of liver alterations during SARS-CoV-2 infection is self-limiting. Concurrent or prevalent drug-induced liver damage can change the evolution of viral-dependent hepatic derangement in the course of COVID-19.

Early diagnosis, fine monitoring and prompt support of patient suffering from hepatic damage during COVID-19 are crucial approaches for its fruitful management.

We do hope that the involvement of the liver during SARS-CoV-2 infection will be remembered in the literature in the next years. Indeed, it is worth description for researchers and clinicians involved in this pandemic.

## Figures and Tables

**Table 1:** Main medications used during COVID-19 treatment associated with mild to severe DILI development.

*Medication*	*Hepatotoxic Mechanism(s) and Damage(s)*	*Reference Number*
**Azithromycin**	Self-limited cholestatic hepatitis, appearing within 1 to 3 weeks after starting treatment. Importantly, cholestasis and increased transaminases can persist for up to 6 months	[47]
**Lopinavir/ritonavir**	Incidence of 3% to 10%; symptoms’ onset (e.g., jaundice) from 1 to 8 week(s) after treatment initiation; range of laboratory tests; hepatocellular to cholestatic or mixed damage. Usually, the liver injury is self-limiting, with a few fatal cases	[48]
**Hydroxychloroquine**	Only if used at high doses can it lead to acute liver injury (namely, sudden onset of fever and marked elevation of serum transaminase)	[49]
**Tocilizumab**	There have been recorded several cases of clinically apparent severe liver injury with jaundice, usually self-limiting, with complete recovery within 2 to 3 months upon drug termination. Indeed, it was the cause of one case of fatal liver failure. Interestingly, registration trials showed serum aminotransferase elevations in 10% to 50% of administered patients	[50]
**Remdesivir**	Ten to 50% of treated patients have shown transient, mild-to-moderate serum transaminases rise within 1 to 5 days of drug initiation. Interestingly, no elevation of serum bilirubin and/or alkaline phosphatase levels was registered	[51]
**Enoxaparin**	They have been described as elevations of serum aminotransferases in 4% to 13% of treated patients. There was a rapid liver damage onset (within 3 to 5 days upon drug administration), and rapid recovery (from 1 to 4 weeks upon treatment discontinuation), with no symptoms. Some patients presented with a mild increase in serum bilirubin and alkaline phosphatase	[52]
**Corticosteroids**	The use of glucocorticoids can result in hepatomegaly and steatosis. Moreover, their use can trigger/worsen non-alcoholic steatohepatitis. Further, long-term administration can reactivate B and C chronic viral hepatitis. Specifically, high doses of methylprednisolone can lead to acute liver damage with fatal acute liver failure. Symptoms account for jaundice (from 2 to 6 weeks after drug discontinuation). Some cases required emergency liver transplantation	[53]

**Table legend:** First line is in bold and italics as it defines medications, hepatotoxic mechanisms and references reviewed; first column is in bold as distinguish medications to be reviewed.

## Data Availability

All the data reviewed in this manuscript are available online. In the specific, they can be retrieved in PubMed, MEDLINE and in the database of the main national and international gastroenterology meetings.
